# Correction: Mitophagy-mediated adipose inflammation contributes to type 2 diabetes with hepatic insulin resistance

**DOI:** 10.1084/jem.2020141606022025c

**Published:** 2025-06-13

**Authors:** Feng He, Yanrui Huang, Zhi Song, Huanjiao Jenny Zhou, Haifeng Zhang, Rachel J. Perry, Gerald I. Shulman, Wang Min

Vol. 218, No. 3 | https://doi.org/10.1084/jem.20201416 | December 14, 2020

The authors regret that, during assembly of their figures, errors were made in Fig. 8 C and Fig. S3 C. A *Trx2*^*ADKO*^ liver BODIPY image from Fig. 3 D was mistakenly used in the *Trx2*^*ADKO*^ saline panel of Fig. 8 C. In Fig. S3 C, the *Trx2*^*ADKO*^/24 wk insulin-only staining image (bottom right panel) was mistakenly used in the WT/6 wk insulin/TUNEL pancreas panel on the top left. These errors do not affect the conclusions of the study, and the figure legends remain unchanged. Both the original and revised Fig. 8 and Fig. S3 are shown here. The errors appear in print and in PDFs downloaded before June 2, 2025.

**Figure:**
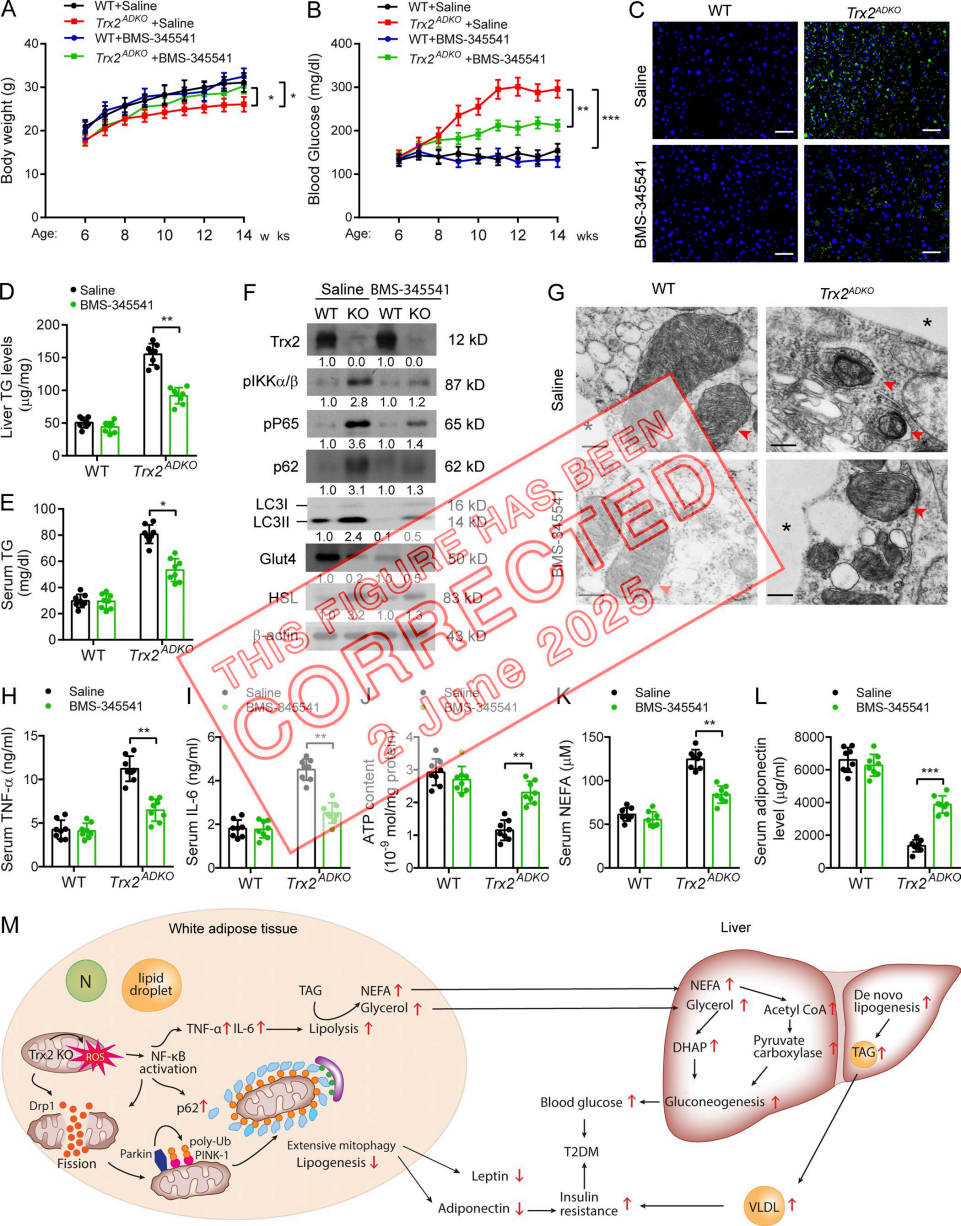


**Figure 8.**
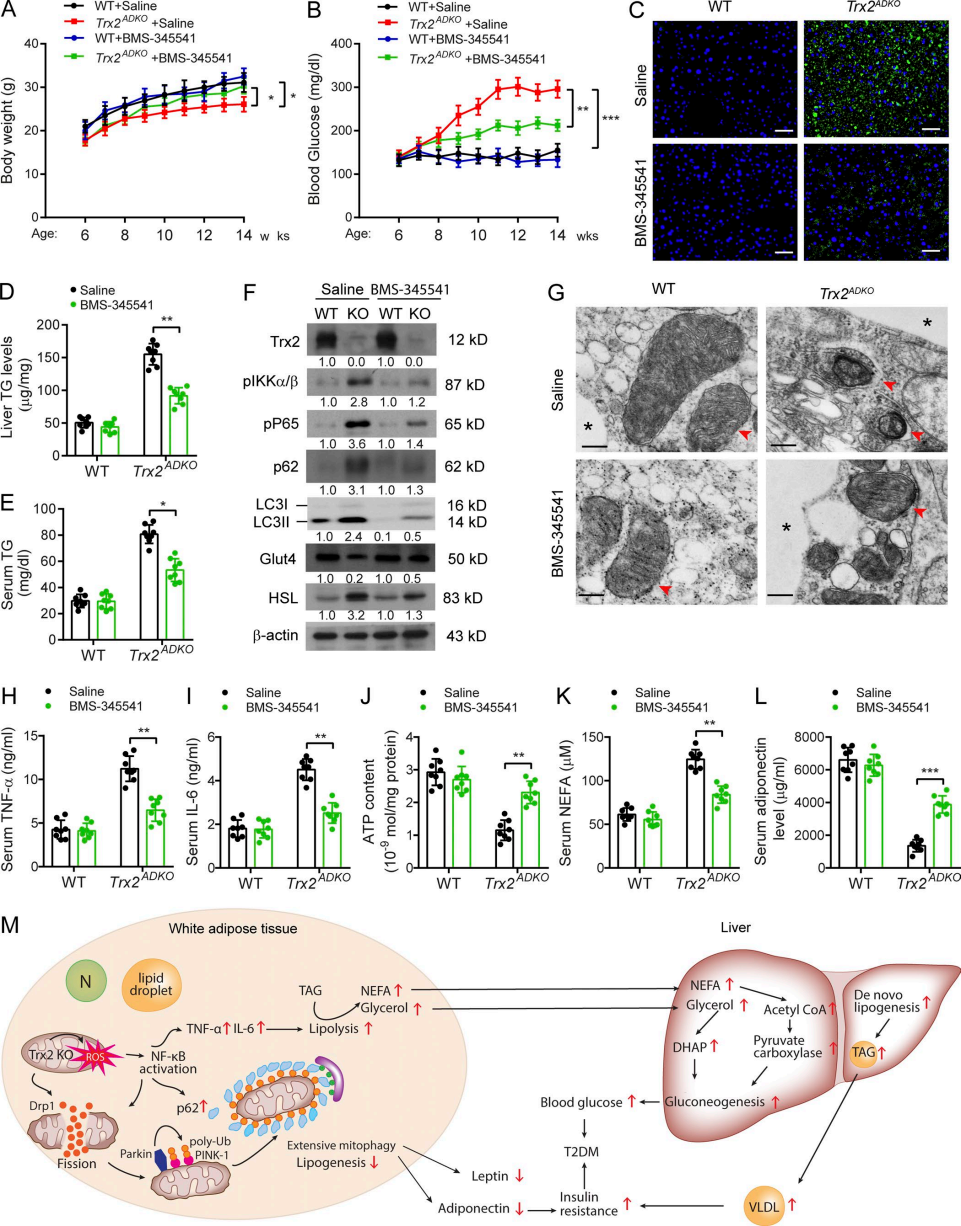
**Inhibition of NF-κB activity ameliorates T2DM in *Trx2*** ^***ADKO***^ **mice.** 6-wk-old male *Trx2*^*ADKO*^ and WT mice were treated with 60 mg/kg BMS-345541 by i.p. injection once every 2 d for 8 wk. **(A and B)** Body weight (A) and fasting blood glucose levels (B) in WT and *Trx2*^*ADKO*^ mice with or without BMS-345541 treatment (*n* = 8) at the indicated ages. **(C)** Representative images of BODIPY staining showing liver lipid deposition of mice at 14 wk of age. Scale bars, 50 µm. **(D and E)** Liver TG content and serum TG level were measured. *n* = 6. **(F)** Immunoblot analysis of eWAT tissues from mice at 14 wk of age. Protein levels were quantified and presented as fold changes by taking WT as 1.0. *n* = 3 mice for each group. **(G)** Representative transmission electron micrographs of eWAT sections from mice at 14 wk of age (six images/mouse, *n* = 3 mice/group). Asterisks indicate LDs. Arrowheads indicate mitochondria. Scale bars, 0.5 µm. **(H and I)** Serum cytokines TNF-α and IL-6 proteins were measured by ELISA kits (*n* = 8). **(J)** ATP content of mitochondria isolated from eWAT of mice at 14 wk of age (*n* = 8). **(K and L)** Serum levels of NEFA (K) and adiponectin (L) of 14-wk-old mice (*n* = 8). Quantitative data are presented as mean ± SEM. *, P < 0.05; **, P < 0.01; ***, P < 0.001 versus the indicated comparisons. Significance was assessed by one-way ANOVA followed by Tukey’s post hoc test. **(M)** A schematic diagram summarizing our findings that Trx2 deficiency promotes severe mitophagy via mitochondrial ROS/NF-κB/p62 signaling, which contributes to hepatic insulin resistance related T2DM (see text for details). N, nucleus; DHAP, dihydroxyacetonephosphate. TAG, triacylglycerol; VLDL, very low-density lipoprotein.

**Figure:**
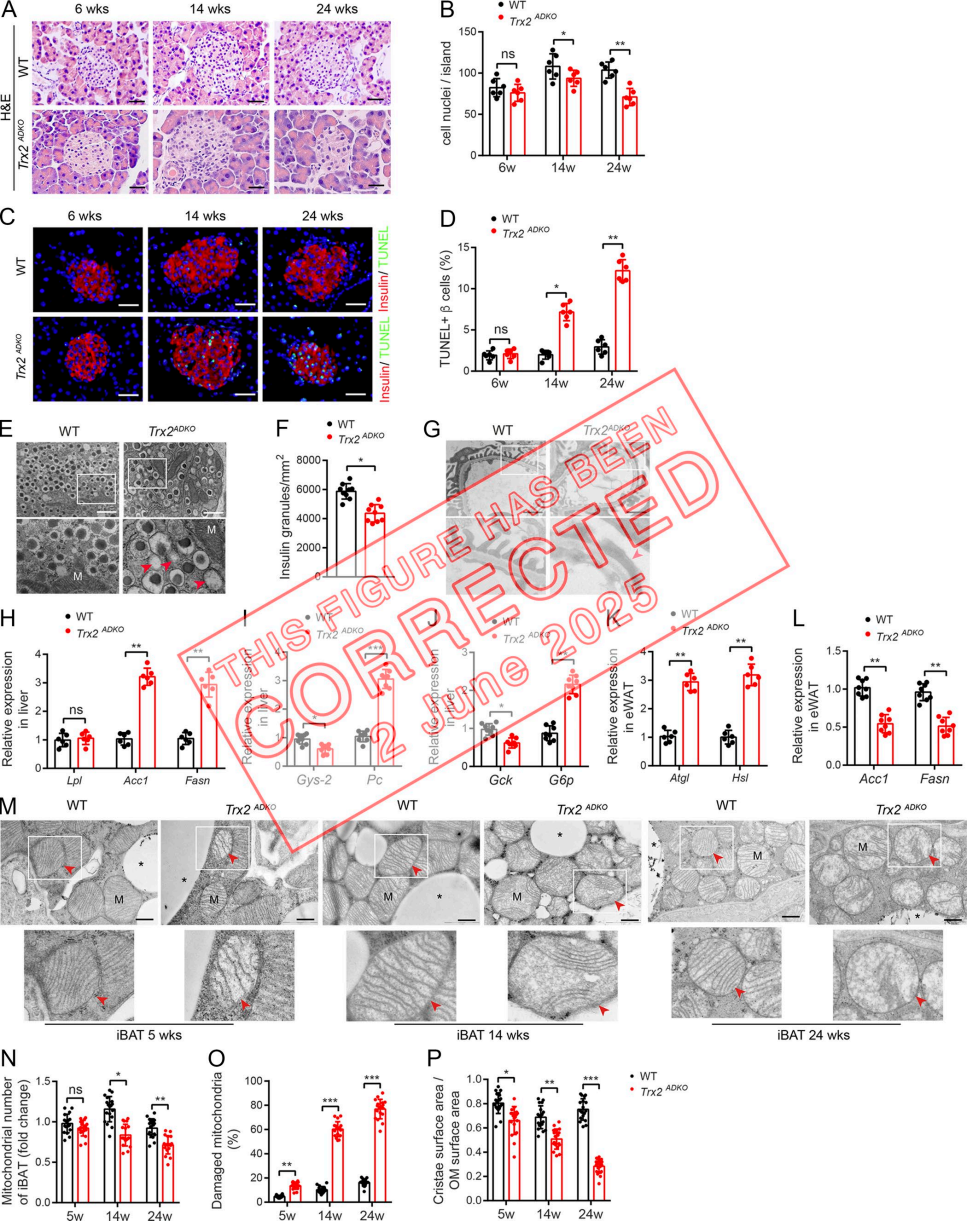


**Figure S3.**
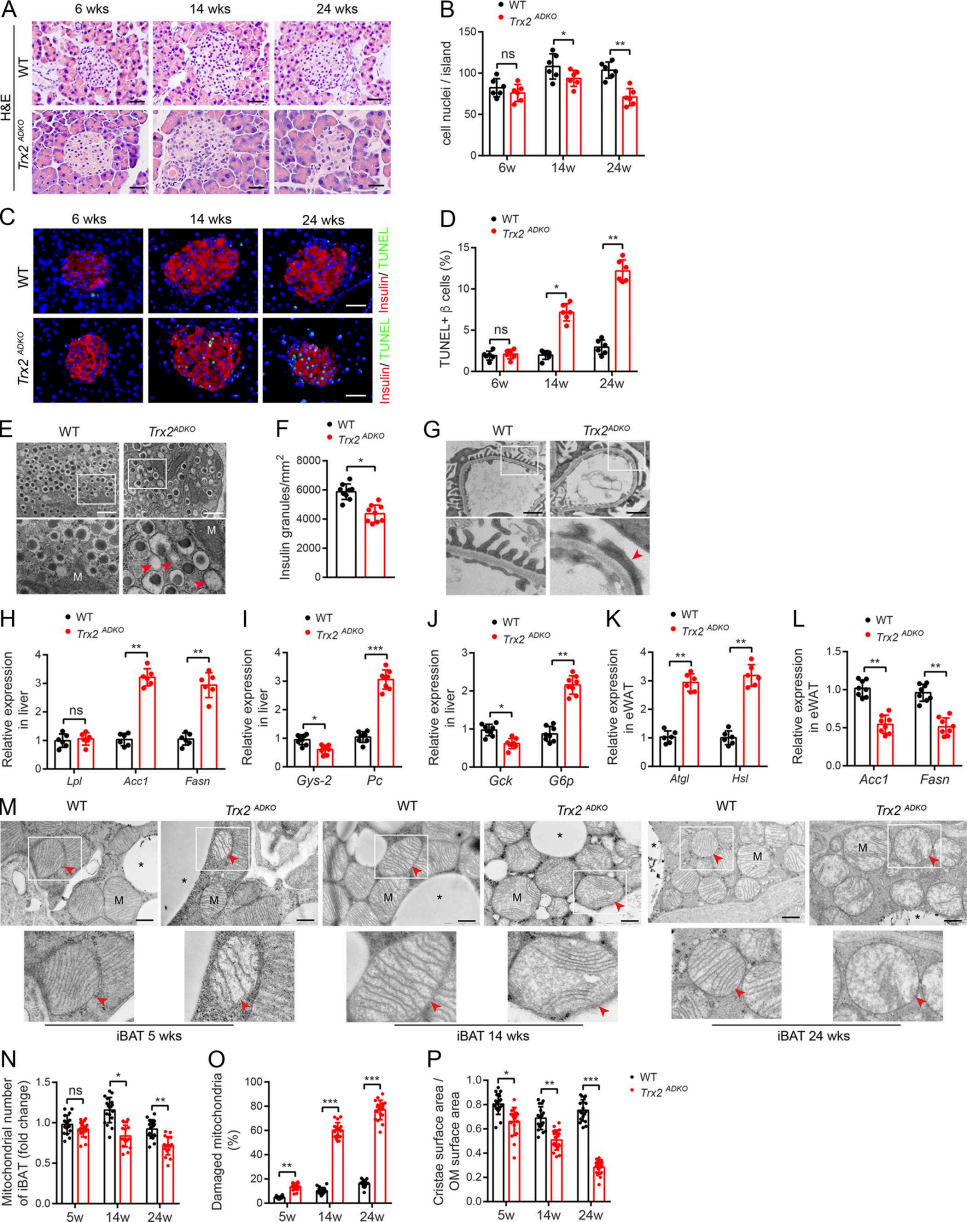
***Trx2*** ^***ADKO***^ **mice develop T2DM-related end-organ damage. (A–E)** *Trx2*-KO mice exhibit decreased insulin content and increased β cell apoptosis. **(A)** Representative hematoxylin and eosin–stained pancreas sections showing pancreatic islets of WT and *Trx2*^*ADKO*^ mice at the indicated ages. Scale bars, 20 µm. **(B)** Nuclei density of six randomly selected pancreatic islets (*n* = 6 mice). **(C)** Detection of β cell apoptosis by costaining of TUNEL (green) and insulin (red). Representative images from WT and *Trx2*^*ADKO*^ mice at the indicated ages. Scale bars, 20 µm. **(D)** Quantification of TUNEL-positive β cells (right panel; *n* = 6 mice). **(E)** Representative transmission electron micrographs of pancreas tissue from WT and *Trx2*^*ADKO*^ mice (three images/mouse, *n* = 3 mice/group). Squares correspond to the magnified areas (bottom panel). Scale bars, 1 µm. M, mitochondria. Arrowheads indicate empty granules. **(F)** Quantification of insulin granules per µm^2^ islet. **(G)** Representative transmission electron micrographs of kidney tissue from WT and *Trx2*^*ADKO*^ mice (*n* = 3). White squares correspond to the magnified areas (bottom panel). Red arrowhead indicates podocyte foot process fusion. Scale bars, 1 µm. **(H–L)** Quantitative analysis of de novo lipogenesis and hepatic gluconeogenic genes. **(H)** Relative mRNA expression of lipogenesis genes in liver in 14-wk-old male WT and *Trx2*^*ADKO*^ mice (*n* = 6). **(I and J)** Relative mRNA expression of hepatic gluconeogenic genes in liver of 14-wk-old male WT and *Trx2*^*ADKO*^ mice (*n* = 8). **(K)** Relative mRNA expression of the indicated de novo lipogenesis genes in eWAT of 14-wk-old male WT and *Trx2*^*ADKO*^ mice (*n* = 8). **(L)** Relative mRNA expression of lipolysis genes in eWAT in 14-wk-old male WT and *Trx2*^*ADKO*^ mice (*n* = 6). Quantitative data represent the mean ± SEM. ns, not significant; **, P < 0.01; ***, P < 0.001 compared with WT controls (two-tailed Student’s *t* test). Acc, acetyl-CoA carboxylase 1; *Atgl*, adipose TG lipase; Fasn, fatty acid synthase; *G6p*, glucose 6-phosphatase; *Gck*, glucokinase; *Gys2*, glycogen synthase 2; *Hsl*, hormone-sensitive lipase; Lpl, lipoprotein lipase; *Pc*, pyruvate carboxylase. **(M–P)** TEM analysis of brown adipose mitochondria. **(M)** Representative transmission electron micrographs of interscapular BAT (iBAT) sections from WT and *Trx2*^*ADKO*^ mice at the indicated ages. Asterisks indicate LDs. Squares correspond to the magnified areas (bottom panel). Arrowheads indicate mitochondria. Scale bars, 0.5 µm. **(N–P)** Number of mitochondria, number of damaged mitochondria, and cristae surface area/outer membrane (OM) surface area (six images/mouse; *n* = 3 mice/group). Quantitative data represent the mean ± SEM. ns, not significant; *, P < 0.05; **, P < 0.01; ***, P < 0.001 versus WT (two-tailed Student’s *t* test). w, weeks.

